# Bacteria Murmur: Application of an Acoustic Biosensor for Plant Pathogen Detection

**DOI:** 10.1371/journal.pone.0132773

**Published:** 2015-07-15

**Authors:** George Papadakis, Nicholas Skandalis, Anastasia Dimopoulou, Paraskevas Glynos, Electra Gizeli

**Affiliations:** 1 Institute of Molecular Biology and Biotechnology, Heraklion Crete, Greece; 2 Benaki Phytopathological Institute, 8 St. Delta, Kifissia, Athens, Greece; 3 Dept. of Biology, University of Crete, Vassilika Vouton, Heraklion Crete, Greece; College of Agricultural Sciences, UNITED STATES

## Abstract

A multi-targeting protocol for the detection of three of the most important bacterial phytopathogens, based on their scientific and economic importance, was developed using an acoustic biosensor (the Quartz Crystal Microbalance) for DNA detection. Acoustic detection was based on a novel approach where DNA amplicons were monitored and discriminated based on their length rather than mass. Experiments were performed during real time monitoring of analyte binding and in a direct manner, i.e. without the use of labels for enhancing signal transduction. The proposed protocol improves time processing by circumventing gel electrophoresis and can be incorporated as a routine detection method in a diagnostic lab or an automated lab-on-a-chip system for plant pathogen diagnostics.

## Introduction

Detection of bacterial plant pathogens relies on internationally agreed diagnostic protocols published by official entities such as the European and Mediterranean Plant Protection Organization [1]. They are based on biochemical tests, serological typing (immunofluorescence, enzyme-linked immunosorbent assay—ELISA, protein profiling (SDS-PAGE), fatty acid methyl-ester (FAME) profiling, pathogenicity confirmation testing) and polymerase chain reaction (PCR)—based techniques [2]. PCR mainly focuses on amplification of the 16S rRNA gene and the 16S-23S internal transcribed spacer by genera or species specific primers, combined occasionally with simple restriction fragment length polymorphisms (RFLPs) and repetitive-sequence-based PCR (REP-PCR) analysis. Other primer targets usually include genera and species specific genes [3]. Real time, multiplex and competitive PCR protocols have been developed [4,5] to overcome main drawbacks of PCR-based methods: sensitivity, cross reaction with other bacteria, and false negatives or positives, usually associated to the DNA extraction method used and/or the plant tissue [6].

In a recent study, the polymerase chain reaction was paired with acoustic biosensors for detecting different types of mutations in Anopheles and the human BRCA1 and BRCA2 genes [7,8]. The described methodology combines the sensitivity and selectivity of the PCR method with the label free nature of acoustic biosensing while DNA detection is achieved within only a few minutes and during real time monitoring. The amplified PCR products can be directly immobilized through biotin-avidin interactions [9] on the surface of the acoustic sensor without the need of post-PCR purification. DNA binding affects the characteristics of the acoustic waves propagating on the sensor surface, i.e. velocity and energy, which in turn are monitored as changes in frequency and dissipation [10]. Frequency changes can be related to the mass of the immobilized DNA while energy dissipation to the viscoelastic properties of the bound molecules. The energy dissipation per unit mass ratio (acoustic ratio) was shown to be related to the length of the attached analyte [7,11].

The current work, combines for the first time the Quartz Crystal Microbalance (QCM), a commercial acoustic biosensor system, with a multiplex PCR reaction for the simultaneous detection of three of the most economically/scientifically important [12], plant bacterial pathogens; i.e. *Ralstonia solanacearum (Rsol)*, *Pseudomonas syringae* pv *tomato* (Pto) and Xanthomonas *campestris* pv. *vesicatoria* (Xcv). All the above which include tomato (*Solanum lycopersicum*) as their host range cannot be accurately detected with conventional PCR methods are results are often inconsistent. For example, most protocols for *Xanthomonas campestris* pathovar detection target the 16S rRNA gene which exhibits a 98% similarity within the genus *Xanthomonas*[13]. To develop the multiplexed assay initially we assessed all the available and approved primers for the detection of the selected three pathogens [3] and selected those that resulted in three products of distinct sizes.

Bacterial wilt is a devastating plant vascular disease caused by *Ralstonia solanacearum*, a genetically diverse soil-borne pathogen with an extremely wide host range [14]. It occurs in tropical, subtropical and warm temperate areas throughout the world, causing great losses in agriculture, is considered a quarantine pathogen and no effective chemical product is available for its control.

*Pseudomonas syringae* is a widespread bacterial pathogen that causes disease on a broad range of economically important plant species. The species *P*. *syringae* is sub-divided into about 50 pathovars, each exhibiting characteristic disease symptoms and distinct host-specificities [15]. *P*. *syringae* pathovar *tomato* (*Pto*) is the cause of bacterial speck of tomato, a seedborne disease that has become economically important throughout the world since the mid-1970s.

Bacterial spot of pepper and tomato plants, is caused by bacterial spot-causing xanthomonads (BSX) of which two distinct groups compose the formerly known Xanthomonas *campestris* pv. *vesicatoria*[16]. *Xcv* is considered a quarantine organism in the European Union A2 list [1] due to its economic importance and its prolong survival in tomato and tomato seeds.

## Materials and Methods

### Bacterial strains, growth conditions and inoculation assays

Bacteria utilized in this study included *P*. *syringae* pv *tomato* Rifampicin resistance type strains DC3000/ DC3001 [17,18] strains BPIC 315 and BPIC 389 from the Benaki Phytopathological Institute culture Collection (BPIC); *R*. *solanacearum* type strain *GM*I 1000 and strain BPIC 819 (BPI) and *Xanthomonas campestris pv*. vesicatoria strain 5071 (D. Goumas, pers. collection) and other *Xcv* strains isolated from pepper and tomatoes at BPI. Strains were routinely grown on LB medium, NA agar [19] for 24 h at 28°C; *Pto* strain were also grown in King's B (KB) medium [20].

To prepare inoculums, cultures were grown in 5 ml of liquid LB medium; bacterial cells were then harvested by centrifugation at 2.800 rpm for 10 min in 10°C, washed twice in 10 mM MgCl_2_ and finally re-suspended to an OD600 of 0.12 (10^8^ cfu ml^-1^ in sterile 10 mM MgCl_2_). Inoculum concentration was then adjusted to 5 ×10^5^ cfu ml^-1^, and used for inoculation of tomato plants cv. Belladonna. Plants were grown from surface sterilized seeds which were germinated on moist potting soil and then transplanted into 10 cm pots containing a three-element (NPK) complex fertilized soil and maintained in a glasshouse at 25°C and 16h photoperiod. They were inoculated at the 4 to 5 true leaf stage following respective schemes for each pathogen. In detail, *Pto* and *Xcv* inoculums were infiltrated by gently pressing a syringe on the abaxial surface of leaves and injecting 150 μl into the intercellular spaces [21]. In addition, to simulate natural infection a 10^8^ cfu ml^-1^ concentration of *Pto* and *Xcv* inoculums-prepared as described above—were also sprayed in the upper and lower surface of leaves in the presence of the X77 (Loveland Inc.) spreading agent. *Rsol* inoculum was also prepared as described above, adjusted to cell density of 10^7^ cfu ml^-1^ and a 50 ml suspension was then drenched to pots containing tomato plants.

### Total cell count determination

Total cell count of each of the bacterial suspensions was determined by plating 100 μl samples of serial 10-fold dilutions of each suspension. The internal pathogen population at each sampling point was calculated using 8 mm-diameter leaf/vascular tissue samples that were surface sterilized for epiphytic bacteria. Samples were then homogenized in 10mM MgCl_2_, before plating the 100 μl samples of the homogenate onto NA agar containing the appropriate antibiotic to allow for colony forming unit (cfu) development after a 48 h incubation at 28°C.

### Isolation and manipulation of bacterial DNA

Genomic DNA from bacterial cultures was extracted based on the method developed by Murray and Thompson (1980)[22]. *Bacterial* DNA was isolated from infected plant tissue either by diffusion of bacteria, centrifugation at 4.000 rpm for 10 min in 10°C and application of the previously mentioned protocol, or as crude DNA extract using Whatman FTA Elute Cards (USA). Samples were collected daily after inoculation.

### Evaluation of multiplex PCR sensitivity

Multiplex PCR sensitivity was evaluated using DNA extracts from bacterial cultures ranging from 10^7^ to 10^1^ cfu ml^-1^. The same range of pathogen suspensions were also used as whole cell templates for PCR amplification as described by Cuppels et al. (2006)[17]. The limit of detection and the interference of tomato tissue with the efficiency of DNA extraction or PCR amplification was also determined in single pathogen infections by extracting bacterial DNA from infected plant tissue samples on a daily basis. The pathogen population on each sample was counted as mentioned previously.

### Multiplex PCR assay

To develop a multiplex PCR for the simultaneous detection of three pathogens using an acoustic biosensor several primer pairs were assessed *in silico* for compatibility in a multiplex PCR. The following PCR primer pairs were selected: OLI1 (5’ GGGGGTAGCTTGCTACCTGCC 3’) and Y2 (5’ cccactgctgcctcccgtaggagt 3’) for amplification of a 288 bp fragment from a *Rsol* DNA template [23]; COR1 (5’ gga ctc agc agt atc atc tcg gga cg 3’) and COR2 (5’ tgc agg gtc ttg ggg agc acg 3’) for amplification of a 689 bp fragment from a *Pto* DNA template [17]; RST 65 (5’ GTC GTC GTT ACG GCA AGG TGG TCG 3’) and RST69 (5’ tcg ccc agc gtc atc agg cca tc 3’) for amplification of a 420 bp fragment from a *Xcv* DNA template [24]. Each primer pair was tested under the reported PCR conditions using genomic DNA or by increasing the denaturation step 3 mins for whole cell templates. Amplification was performed in 0.2 ml thin-walled PCR tubes in a Applied Biosystems (USA) Veriti thermal cycler. The final 25 μl reaction mixture contained 1 x PCR buffer, 2.0 mM MgCl_2_, 0.2 mM of each dNTP, 0.8 μM each primer and 1U of KAPATaq DNA polymerase (Kapabiosystems, USA) in addition to 1μl of bacterial DNA template.

Once end-point PCR was standardized, a multiplex reaction protocol was developed using gradient PCR, seeking to amplify products from DNA, whole cell or infected plant material templates. Gradient PCR was performed in the following conditions: one cycle at 95°C for 2 mins (5 mins for whole cell template); 30 cycles of 30 sec at 95°C, 30 sec at 54, 58, 61, 63, 66 or 68°C and 40 sec at 72°C; and final extension at 72°C for 5 mins. Each single PCR product from multiplex PCR was sequenced on sense and antisense strands using an ABI 3730 sequencer. To increase the speed of the multiplex amplification protocol the KAPA2G Fast HotStart Taq DNA polymerase (Kapabiosystems, USA) was employed using the following cycling conditions: one cycle at 95°C for 2 mins (5 mins for whole cell template); 30 cycles of 10 sec at 95°C, 15 sec at 63°C and 10 sec at 72°C. The two end-point multiplex PCR protocols described above were repeated using 5’-end biotinylated (FRIZ, Germany) and non-biotinylated primers as forward and reverse primers, respectively. The PCR products (5μl) were loaded into 1.5% agarose gels in 0.5 TBE buffer (O.089M Tris-borate, 0.002M EDTA, pH 8.0) for electrophoresis and analysis under standard conditions. Products that were amplified using the fast-multiplex PCR protocol were used for the acoustic measurements.

### Experimental setup and real time acoustic measurements

**Fig 1:**
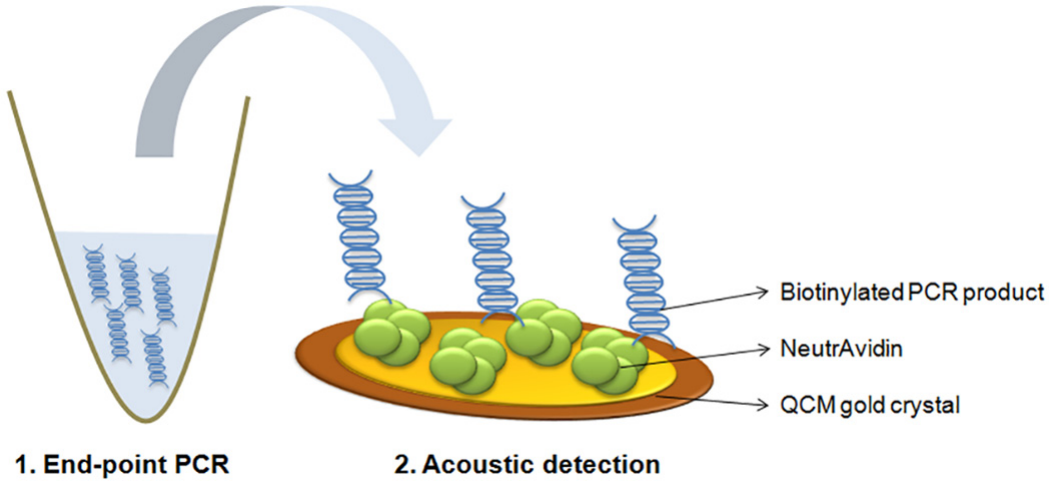
Experimental procedure for acoustic measurements. The method consists of two steps: 1. End-point PCR and 2. PCR product immobilization. Before addition of the PCR reaction, neutravidin protein is adsorbed on a clean gold QCM crystal and biotinylated DNA is immobilized through biotin-neutravidin interactions. Biomolecules are not drawn in scale.

Acoustic measurements were performed with the Q-Sense E4 instrument (QSense, Sweden) using gold crystals operating at 35 MHz, cleaned by etching for 2.30 min at high power in a Harrick Plasma Cleaner using air. The gold sensors were immediately transferred to the instrument and the system was filled with PBS buffer using a peristaltic pump at a flow rate of 75 μl/min. 200 μl of neutravidin (Invitrogen) at a concentration of 200 μg/ml were adsorbed at the sensor surface followed by PBS rinsing. PCR reactions without purification were diluted in PBS buffer to a volume of 200 μl and loaded on the sensor followed by buffer rinsing (Fig 1).

Real time graphs of dissipation (D) and frequency (F) during DNA binding were used to measure the acoustic ratio expressed as ΔD/ΔF, where ΔF corresponds to the raw frequency data, i.e. not divided by the overtone number. Multiplex fast PCR reactions containing template corresponding to each of the 3 pathogens and one non template control (NTC) (negative) reaction without template were prepared and loaded on the sensor. The reactions were added on the same sensor in a consecutive order. Firstly, (A) the negative control followed by the 3 PCR reactions containing amplicons corresponding to (B) *Pto*, (C) *Rsol* and finally (D) *Xcv* pathogens. Between samples addition the sensor was rinsed with PBS buffer. Each measurement was repeated three times. Additions in a consecutive order is possible since it has been shown that the acoustic ratio is independent of the surface history, i.e., previous loading steps [11,25], as long as the available binding positions have not reached saturation.

## Results

### Development of a multiplex PCR for acoustic detection

The acoustic method for DNA detection is achieved during the immobilization of biotinylated DNA molecules on a neutravidin coated device surface (Fig 1). DNA binding causes changes in the frequency (ΔF) and dissipation (ΔD) of the acoustic wave. Acoustic measurements are then expressed as an acoustic ratio (ΔD/ΔF) which is linearly related to the number of base pairs of a DNA molecule, approximately up to 300 bp [7]. Above this linear range, the acoustic ratio still increases with DNA length up to 1000 bp at which point a plateau is reached (data not shown). Towards the development of a multiplex assay for the simultaneous detection of 3 different pathogens with the acoustic method, the primer pairs should be chosen so that the produced PCR amplicons are between 100 and 1000 bp and with differences of at least 100 bp between any two of them. This difference in base pairs number was considered essential to ensure that three clearly distinct acoustic ratios corresponding to three distinct DNA lengths would be measured with the acoustic sensor.

**Fig 2:**
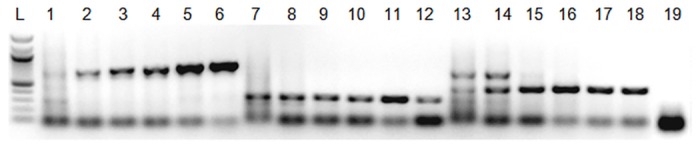
Multiplex polymerase chain reaction showing specificity of three primer pairs to amplify *Pseudomonas syringae* pv. *tomato* (lane 1–6), *Xanthomonas campestris pv*. *vesicatoria* (lane 7–12) or *Ralstonia solanacearum* (lanes 13–18) at anealling temperatures of 58, 61, 63, 66, 68, 69°C; lane L: 1 kb ladder; lane 19: non-template control.

Several primer pairs were assessed *in silico* for pairwise compatibility in a multiplex PCR for the simultaneous detection of *Rsol*, *Pto* and *Xcv* (data not shown). The selected primer pairs were OLI1 and Y2, amplifying a 288 bp fragment of the 16S rRNA gene in the case of *Rsol*[23]; COR1/2, amplifying a 689 bp fragment of a gene cluster controlling production of the *Pto* phytotoxin coronatine [17]; and RST 65/69, amplifying a 420 bp fragment of the *Xcv hrp* gene cluster, encoding for a protein secretion system [26], for [24]. An end point multiplex PCR was developed using gradient PCR; amplification was specific at an annealing temperature of 63–69°C (Fig 2) and specificity was confirmed by sequencing of the amplified products on sense and antisense strands. Amplification was successfully tested for each of the three pathogens and in DNA extracts of overnight cultures, whole cell templates and infected tissue (crude extracts or diffused bacteria).

### Acoustic detection of the 3 pathogens

**Fig 3:**
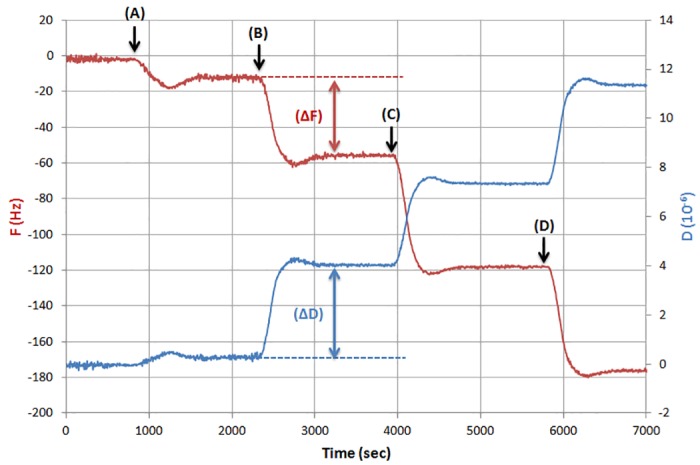
Real time graph depicting changes in dissipation and frequency upon samples addition in a consecutive order. (A) Non template control, (B) *Pseudomonas syringae* pv. *tomato*, (C) *Ralstonia solanacearum* and (D) *Xanthomonas campestris* pv. *vesicatoria* multiplex PCR reactions.

**Fig 4:**
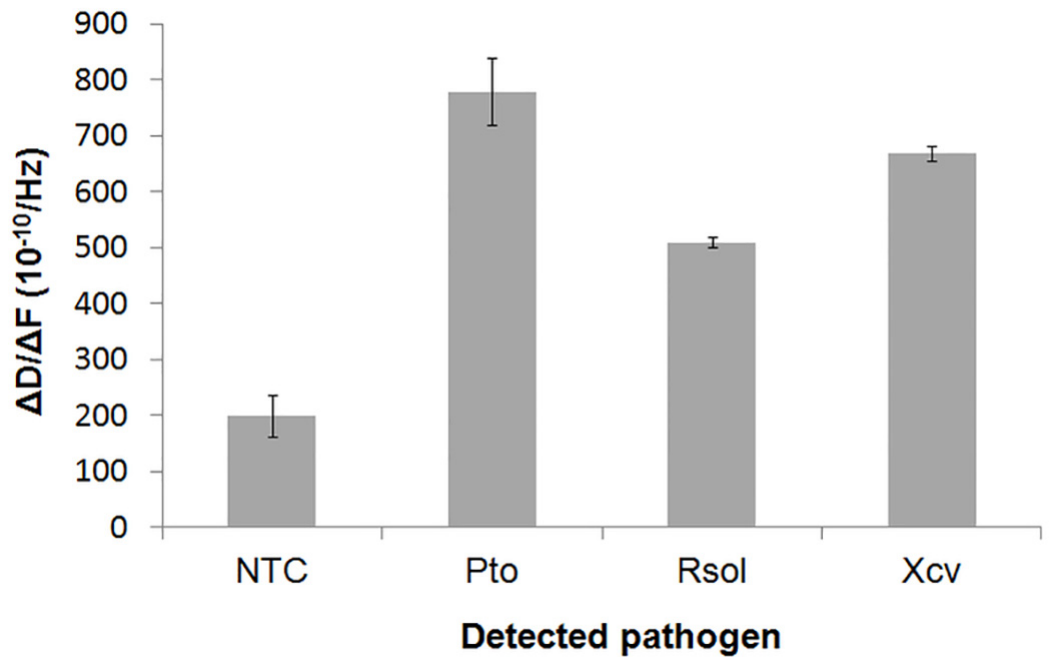
Comparison of the acoustic ratios measured for the 3 different pathogens and the negative control.

Real time binding graphs (Fig 3) were used to calculate the acoustic ratio (ΔD/ΔF) for every binding step, each one corresponding to the addition of one of the target DNAs; the acoustic ratios obtained for each pathogen are summarized in fig 4.

The acoustic ratio for the NTC reaction 199±38 (10^−10^/Hz) is in agreement with previous data from similar measurements of unpurified PCR reactions with QCM-D [8]. When DNA template was added to the multiplex reaction three distinct acoustic ratios were measured. For the 689 bp amplicons corresponding to *Pto* the acoustic ratio was 778±59 (10^−10^/Hz), while for the 288 bp of *Rsol* and the 422 bp for *Xcv* it was found to be 509±8 (10^−10^/Hz) and 669±13 (10^−10^/Hz), respectively. All three ratios are significantly above the corresponding control value and since no overlapping is observed among them, each one can be clearly attributed to a particular pathogen (Fig 4). Note that ratios correspond to the mean value derived from at least 3 experiments.

### Limit of detection of the multiplex PCR and the acoustic detection method

When DNA extracts from pure bacterial cultures were used as templates for the multiplex PCR reaction and amplified products were visualized in agarose gels, the limit of detection was found to be 10^1^−10^2^ cfu with each bacterial DNA template. When whole bacterial cell were used as templates, the limit of detections was found to be 10^2^−10^3^ cfu. Therefore, sensitivity levels for the multiplex PCR do not differ to those reported for the respective single PCR or similar multiplex PCR protocols [27].

**Fig 5:**
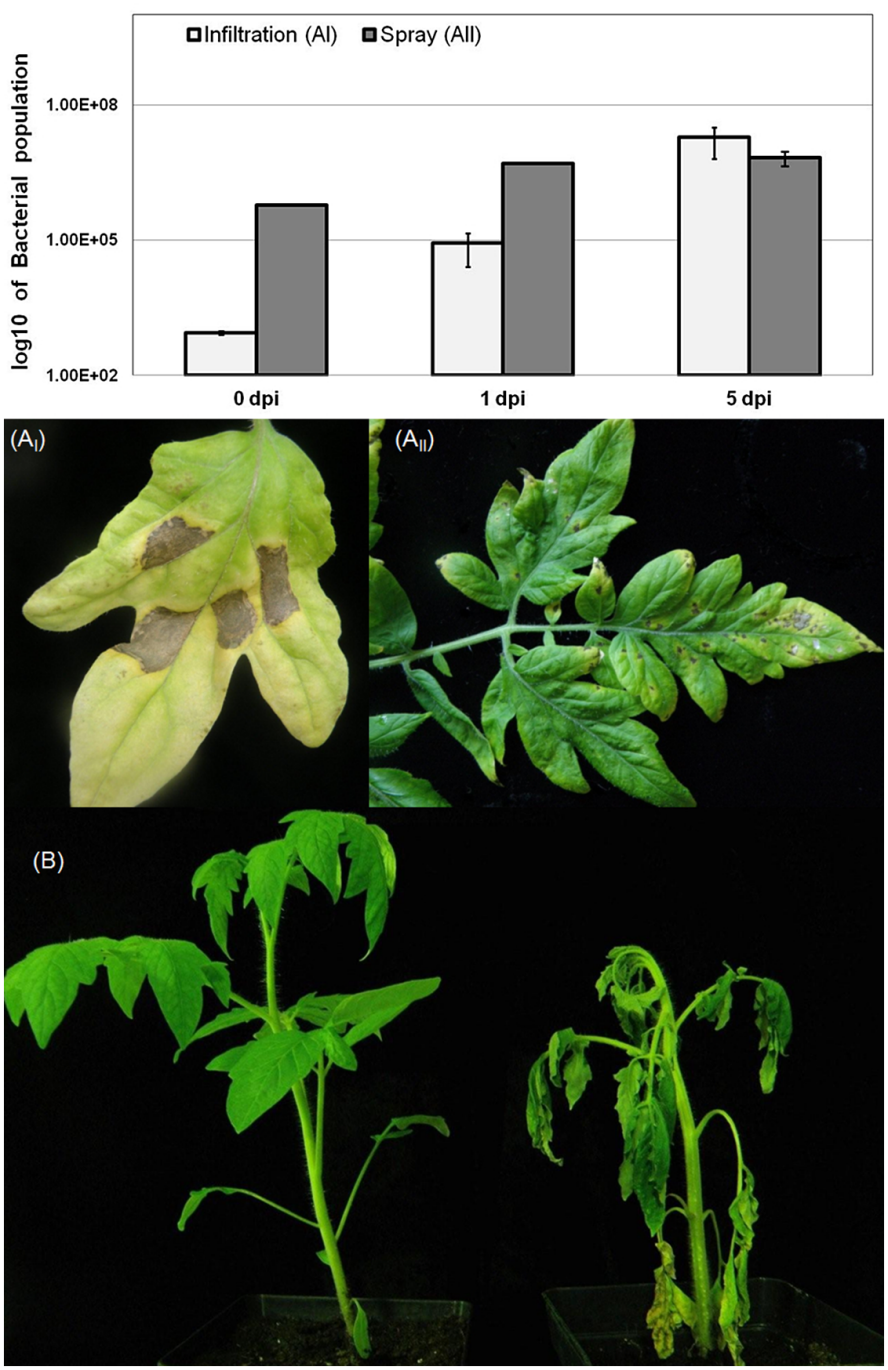
Pathogen population growth and disease symptoms in tomato plants grown to the 4–5 true leaf stage (BBCH 104–105) following artificial infection (inoculation) with A: *Pseudomonas syringae* pv *tomato (Pto)* suspension which was: I. Infiltrated at a 5 ×10^5^ cfu ml^-1^ concentration inside the leaf apoplast using a syringe, developing a necrotic lesion 4 dpi **II**. Sprayed at a 10^8^ cfu ml^-1^ concentration on both sides of the leaf, developing indistinct dark, round spots (specks) after entry through the stomata and epidermal wounds 7 dpi. The increase in *Pto* population size was determined in leaf discs (3 triplicated samples) and over a 17 day time course following treatments and infection. Bars represent means ± SE in log10 scale; *dpi*: days post inoculation. **B**. *Ralstonia solanacearum* suspension drenched to pots containing tomato plants in the 4-5^th^ true leaf stage. Early symptoms appear in the youngest leaves having a flabby appearance followed by wilting of the whole plant 7 days post inoculation (dpi) due to invasion of xylem vessels and collapse of the vascular system.

In *Rsol* and *Pto* single pathogen infection assays, the extraction of crude DNA directly from inoculated plant tissue was found to be more efficient compared to diffusion of bacteria from tissue followed by DNA extraction. In detail, the limit of detection with crude DNA extracts as templates was found to be 10^3^−10^4^ cfu. This corresponds to a bacterial population of 10^6^ cfu/cm^2^ of plant tissue, in samples taken 2 days post infiltration (Fig 5AI) or 6 days after spraying with *Pto*, and before the appearance of tissue collapse and development of necrotic lesions (Fig 5AII). Similarly, detection limits correspond to samples taken 7 days after *Rsol* inoculation, when wilting symptoms appeared (Fig 5B). The method was also successfully tested in DNA extracts of overnight bacterial cultures and inoculated plant tissue using the fast PCR version of the same protocol at an annealing temperature of 63°C.

Regarding the acoustic detection, DNA extracted based on the above methods can be used in the reposrted assay. The amount of PCR amplicons required to produce a reliable acoustic signal change should be in the range of 10–100 ng after end-point PCR. Experiments with extracted DNA from bacteria have shown that the acoustic biosensor can be used to detect PCR products derived from approximately 5 genome copies included in the amplification reaction (unpublished data).

## Discussion

In the current work a multiplex PCR assay was developed for the first time for the detection of three economically important plant pathogens using an acoustic biosensor. Plant pathogens develop symptoms that can often be confused with those of other diseases; additionally, conventional PCR detection protocols sometimes show inconsistency due to pathogen heterogenicity, rendering multi-targeting protocols more efficient for discrimination. The use of acoustic biosensors allows multi-targeting and displays some clear advantages over traditional detection methods. First, acoustic detection can be an attractive alternative to optical sensing since it does not require any fluorescent labels for signal transduction neither the use of elaborate optical instrumentation; In that sense, the development of a real-time PCR instrument for on-site analysis would require at least a light source (LED) for dye excitation, series of filters and lenses to eliminate excitation light and a photomultiplier tube for the detection of the emission light [28]. On the contrary, for acoustic measurements a simple analyzer used for checking antennas and RF circuits would be enough. Second, toxic, genotoxic or other hazardous chemicals are not required as with gel electrophoresis or alternative detection methods, which make the method faster and more user-friendly. Third, double stranded PCR products are directly used for detection instead of hybridizing single stranded DNA molecules to complementary oligonucleotides commonly used with QCM and SPR sensors [29,30]. As a result, the need of controlling hybridization specificity through careful control of the density of surface immobilized probe and hybridization temperature is avoided. Fourth, acoustic detection is very simple since a PCR reaction can be directly loaded on the sensor without the need of purification steps while the measurement is monitored in real time and can be completed within a few minutes. Finally, the sensitivity of the acoustic method combined with the multiplex PCR allows the detection during infections which are latent or in their early stages, before symptom development. Early detection is crucial for emergency responses, biosecurity and microbial forensics of plant pathogens, including *Rsol*, *Pto* and *Xcv*, which are opportunistic pathogens spending part of their life cycle as rhizosphere/epiphytic colonisers of different plants rather than in an active virulent phase of susceptible crops [31]. In addition, this method could assist on-site large scale surveys on random samples where sensitivity, to overcome the problem of false negative results, and fast detection are needed.

In this work the QCM system was employed as a commercially available and well tested instrument for biological and biophysical studies [32]. However, acoustic systems based on surface acoustic wave (SAW) devices would be more suitable for this type of application. Recently, several applications based on SAW devices have been reported for molecular diagnostics, including both protein [33–35] and genetic [36] biomarkers. SAW devices operating at higher frequencies can be easily reduced in size and integrated with microfluidics technology [37,38] to create automated lab-on-a-chip (LoC) platforms for plant pathogen diagnostics. Clear advantages of an acoustic LoC would be the: a) low manufacturing costs; SAW acoustic devices are relatively inexpensive since they are produced in large quantities annually due to their application in mobile phones as bandpass filters [39], b) low energy consumption (battery operated and environmental friendly) and c) portability. Miniaturization and portability will allow ‘on site’ rapid detection, critical for disease control and necessary for detection in remote areas. In addition it could be cost effective and could shorten the time flowchart in large applications, as for example in detection surveys for quarantine pathogens such as *Rsol*. The use of a fast-dependable first confirmation test by phytosanitary inspectors would increase the overall number of samples but decrease the number of samples sent to centralized detection facilities, only to those needed for a second confirmation test.
